# Artificial intelligence applied to fetal MRI: A scoping review of current research

**DOI:** 10.1259/bjr.20211205

**Published:** 2022-03-16

**Authors:** Riwa Meshaka, Trevor Gaunt, Susan C Shelmerdine

**Affiliations:** 1 Department of Clinical Radiology, Great Ormond Street Hospital for Children NHS Foundation Trust, Great Ormond Street, London, UK; 2 Department of Radiology, University College London Hospitals NHS Foundation Trust, London, UK; 3 UCL Great Ormond Street Institute of Child Health, Great Ormond Street Hospital for Children, London, UK; 4 NIHR Great Ormond Street Hospital Biomedical Research Centre, 30 Guilford Street, Bloomsbury, London, UK; 5 Department of Radiology, St. George’s Hospital, Blackshaw Road, London, UK

## Abstract

Artificial intelligence (AI) is defined as the development of computer systems to perform tasks normally requiring human intelligence. A subset of AI, known as machine learning (ML), takes this further by drawing inferences from patterns in data to ‘learn’ and ‘adapt’ without explicit instructions meaning that computer systems can ‘evolve’ and hopefully improve without necessarily requiring external human influences. The potential for this novel technology has resulted in great interest from the medical community regarding how it can be applied in healthcare. Within radiology, the focus has mostly been for applications in oncological imaging, although new roles in other subspecialty fields are slowly emerging.

In this scoping review, we performed a literature search of the current state-of-the-art and emerging trends for the use of artificial intelligence as applied to fetal magnetic resonance imaging (MRI). Our search yielded several publications covering AI tools for anatomical organ segmentation, improved imaging sequences and aiding in diagnostic applications such as automated biometric fetal measurements and the detection of congenital and acquired abnormalities. We highlight our own perceived gaps in this literature and suggest future avenues for further research. It is our hope that the information presented highlights the varied ways and potential that novel digital technology could make an impact to future clinical practice with regards to fetal MRI.

## Introduction

The inherent benefits of magnetic resonance imaging (MRI) make it an attractive modality for fetal imaging as a problem solving, treatment planning or prognostication tool when the limits of obstetric ultrasound are exceeded. It provides excellent soft tissue contrast resolution, improved visualisation through partially ossified structures such as the skull and spine, a larger field of view than obstetric ultrasound, and it is less limited by maternal body habitus and oligohydramnios. Fetal MRI however does have several major limitations. These include the increased time (compared to MRI of other body parts) to obtain diagnostically useful images of a moving target, the limited number of fetal MRI specialists to perform and interpret the imaging, and the time-consuming analysis of multiple sequences and images, often not obtained in perfect planes for biometric measurements. The application of artificial intelligence (AI) for a more automated, reproducible, and time-saving approach to fetal MRI is therefore appealing.^1^ ^2^ ^3^ ^4^

The main aim of this bibliometric scoping review article is to provide readers with a taste of how fetal MRI imaging in the future may benefit from current cutting-edge digital developments and to stimulate avenues for research.

## Methods

The PRISMA extension for scoping reviews (PRISMA-ScR) protocol was used to guide this scoping review, where applicable.^5^ The scoping review was not registered with any online database.

The literature review was performed by two investigators using appropriate prespecified research terms relating to ‘fetus’, ‘MRI’ and ‘artificial intelligence’ (see Supplementary Material 1) within the bibliographic database of MEDLINE (PubMed).

Supplementary Material 1.

The literature search was first performed on 16 September 2021, and an updated repeat search of the literature was again performed on 26 January 2022 using the identical search terms, There were no predefined date limits for the literature review, nor any restriction on sample size. Inclusion criteria were for only peer reviewed papers in English language. Articles which did not use fetal MRI imaging data (*i.e.,* ultrasound imaging) were excluded. Case reports, review articles and opinion pieces were also excluded from the results, although their reference lists were manually interrogated for any important additional references. Grey literature was searched by using the Google search engine and ‘Google Scholar’ database for organisational or societal documents describing the use of AI/ML for fetal imaging.

In order to demonstrate the breadth and variety of how AI/ML were being used for fetal MRI, we did not exclude any papers based on the type of neural network or computational systems employed, nor by the ‘use cases’ described.

Due to the expected heterogeneity of our results and relatively small number of publications, we did not have any predefined tools for bias assessment or quality appraisal, and did not intend to reject any studies on this basis at scoping stage. It was also decided a priori that statistical analyses of the literature would be inappropriate, therefore a descriptive review of our findings was intended from the outset.

## Results

In total, 154 publication titles and abstracts were reviewed. Of these 39 met our inclusion criteria (36 from our initial search, with another three articles included after an updated search), leaving 39 articles for inclusion (see Supplementary Material 2 for data flowchart). All included articles were all published between 2016 and 2021.

Supplementary Material 2.

Nearly two thirds of the articles (*n* = 23, 59%)^6–28^ were published in non-clinical journals (computer science, data science and engineering journals), with the remainder published in more clinical and radiologically targeted journals (*n* = 16, 41%).^29–44^ The vast majority described using AI for imaging the fetal brain (*n* = 26, 67%),^11–22,28,30–41,44^ and a minority for the fetal body (*n* = 5, 13%),^6–9,43^ placenta (*n* = 6, 15%)^23–27,42^ or both (*n* = 2, 5%).^10,29^

The ‘use cases’ for AI in fetal MRI imaging were broadly classified into several main categories, and a selection of the most clinically relevant papers are expanded upon in more detail in the text below:

- Image pre-processing:
  - Dynamic motion correction (*n* = 8, 21%)^7–9,14,17,19,22,28^
- Image post-processing:
  - Segmentation of anatomy (*n* = 16, 41%),^6,12,13,15,16,20,21,24,26,27,29,32,34,36,37,40^
  - Automated fetal biometry measurement (*n* = 1, 3%),^11^
  - Texture analysis (*n* = 1, 3%),^33^
  - Classification of image quality (*n* = 1, 3%)^39^
- Data interpretation:
  - Classification of disease (*n* = 3, 8%),^18,30,31^
  - Prognostication of outcomes (*n* = 4, 10%),^23,41–43^
  - Gestational age prediction (*n* = 2, 5%),^38,44^
  - Generation of clinical 3-D models (*n* = 1, 3%)^25^
- Miscellaneous:
  - Generation of synthetic data (*n* = 2, 5%)^10,35^

### Image Pre-processing

Motion-correction, pre-processing tools provide promising solutions to one of the major challenges in fetal imaging; obtaining high quality images in a constantly moving target. This traditionally required a trained technician to repeatedly adjust acquisition planes and re-acquire sequences. This is time intensive and prone to interoperator variability. Protracted scan times can also be difficult to tolerate for pregnant patients lying still in an enclosed MRI scanner. Automated and accurate correction for fetal motion during initialisation could mean higher quality images and a potentially reduced scan time.

Xu et al^7^ describe a deep learning algorithm, that automatically detects fetal landmarks (using 15 key points – upper limb and lower limb joints, eyes and bladder) to estimate fetal pose which would allow tracking of the fetus and potentially allow for automated readjustment of parameters thus saving technician time and repeated acquisitions during MRI. Their model was trained on 70 3T MRI examinations (fetal gestation 25–35 weeks) using multislice, single shot gradient echo EPI sequences with slice thickness of 3 mm over 10–30 min per examination. They compared the mean error between the predicted fetal pose and position with those of the actual fetal pose acquired (ground truth) and found their model could predict fetal pose to the nearest 4.5 mm in less than 1 sec. . Hou et al^14^ similarly describe a theoretical method of fetal motion correction using a pre-processing AI algorithm (called SVRnet) to predict a 3-D shape orientation of the fetal brain and produce 2-D *T_2_*-weighted single slices at different orientations compensating for motion during initialisation. Singh et al^22^ describe a neural network algorithm which was able to predict fetal motion to within 8 degrees of error, and help estimate slices corrupted by motion to help plan further acquisitions.

In order to improve image quality, Gagoski et al^28^ developed a CNN which performed automated image quality assessment to detect artefacts on T2 HASTE sequences during fetal MRI. They proposed a method where each image slice within an acquisition would be evaluated by the CNN, and at the end of the study, only those slices with the lowest image quality scores would be reacquired by the MRI scanner. This could potentially save time in practice by only reacquiring motion degraded images in clinical practice, instead of a complete new image stack. The study was performed on 10 pregnant mothers with normal fetuses, who underwent repeated HASTE sequence imaging for approximately 50 runs with a reported accuracy of 85.2% achieved (in correctly determining slices that needed to be reacquired).

There was one common limitation for several of these studies – all were at early stages of development and describe the feasibility of a particular pre-processing technique with potential pipeline for deployment, but none demonstrated whether such applications resulted in tangible changes in patient outcome or accuracy in diagnoses. Including these as outcome measures for future studies would be a logical next stage for testing the AI models and an interesting avenue for future research.

### Image Post-processing

Artificial intelligence algorithms have a role in reproducible and time efficient tissue segmentation. The fetal brain has been a popular focus of recent works. Traditionally, manual delineation of the fetal brain on 2D images have been used to produce 3D reconstructions. Li et al^40^ demonstrate the training, validation and testing of a U-net (a convolutional neural network (CNN) developed for medical imaging segmentation) based brain extraction algorithm to automatically segment normal fetal brains on 5-mm slice fetal MRI in three planes. These images were acquired from a 1.5T MRI scanner on either T2 single shot fast spin echo (ssFSE) or balanced steady-state free precession (bSSFP) sequences. The fetal brain segmentation took 2–3 s per brain to complete, compared to 30–40 min by a technician, and achieved a high average Dice coefficient of 0.97 across the three plane. The Dice coefficient is a marker of how much area of overlap there is between two structures (*i.e.,* the actual manual segmentation and predicted segmentation in this case), with a score of 1.0 being a perfect overlapping match.

**Figure 1.**
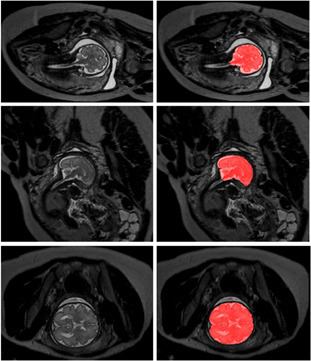
An example demonstrating the use of a U-net to automatically segment the fetal brain from a fetal MRI study. *T*_2_-weighted coronal (top), sagittal (middle) and axial (bottom) images from a fetal MRI scan acquired with a 1.5 Tesla MRI are shown (left), with corresponding automated brain segmentation in red shading overlay (right). Reproduced from Khalili et al^37^, 2019.

Khalili et al^37^ described the successful use of the same U-net algorithm to segment a more diverse set of fetal brains with and without pathology such as intraventricular haemorrhage and stroke, as well as fetal brain images degraded by artefact (Figure 1). Accurate fetal brain measurements of biparietal (BPD) and transcerebellum diameter (TCD) can also be performed by a CNN^11^ which ordinarily are performed manually. Avisdris et al^11^ reported that their algorithm’s mean difference for BPD was 1.45 mm and TCD was 1.23 mm (compared to 1.27 mm and 0.97 mm for two human readers) based on 214 MRI volumes of fetal brains.

Ebner et al^32^ also proposed a framework for automated fetal brain reconstruction based on a four step process using convolutional neural networks to localise, segment and perform super resolution reconstruction. The findings were validated against normal fetal brains and those with varying degrees of pathology, and showed that the automated process was comparable to more labour-intensive, manual segmentations by technicians and radiologists.

**Figure 2.**
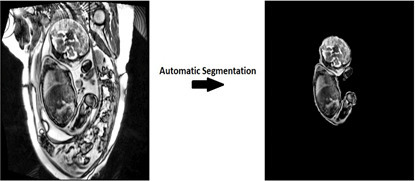
Example of how an automated segmentation algorithm (‘CASE-Net’) could be used to help extract only images of the fetal body from an intrauterine fetal MRI study. Reproduced from Lo J. et al^6^, 2021

Fetal whole-body image extraction on MRI has been proposed to be useful in growth monitoring, pathology detection and more recently, fetal surgical planning.^45^ Only a few whole-body segmentation algorithms in fetal MRI have shown promising results. Lo et al^6^ demonstrated the use of a “Cross-Attention Squeeze Excitation Network” (CASE-Net) (Figure 2) to segment the fetal body (from 1.5T and 3T MRI studies, using T2 sequences) with a DICE coefficient of 0.87. Torrents-Barrena et al^29^ took this topic further by comparing a variety of neural networks in their ability to segment different maternal and fetal body parts on an intrauterine MRI study (uterus, umbilical cord, placenta, lungs, brain) with DeepLabV3+outperforming U-net, V-net and other CNNs in the fetal segmentation tasks. They propose that the ability to automatically segment fetal body parts could be used to aid pre-surgical planning, patient education and consent (*e.g.,* by using the segmentation to create 3D-printed models) or to aid future radiomic studies and texture analysis for predicting outcomes for congenital diseases.

Placental segmentation has been reported to be useful to identify and quantify pregnancy related complications such as placenta accreta or growth restriction.^42,46^ Shahedi et al^24^ describe the use of a U-net based CNN to segment the uterus and placenta in 100 pregnant females to accuracies (*i.e.,* DICE coefficients) of 0.92 and 0.82, respectively. This required minimal user input (reportedly seven ‘clicks’) to produce the output placental size and location.

### Data interpretation

There are relatively fewer studies dedicated to classification of disease and prognostication in fetal MRI as these tasks are more complex and require large datasets of rare fetal pathologies for training. Nevertheless, early studies are promising - for example Attallah et al^18,30,31^ use a publicly available dataset^47^ of 225 fetal MRIs of varying gestational age (16–39 weeks, 113 normal, 114 abnormal fetal MRIs comprising of 21 types of neurodevelopmental disorders) to test a CNN in identifying brain abnormalities. This yielded an accuracy of 95% in differentiating normal from abnormal fetal brains, with abnormalities that included pathologies such as Dandy-Walker spectrum malformations, colpocephaly, agenesis of the corpus callosum and polymicrogyria. Whilst these specific pathologies would be easily recognised by radiologists familiar with interpreting fetal MRI studies, the use of this AI tool would not necessarily provide additional useful diagnostic information but may be useful in triaging which MRI studies require urgent review over the normal cases.

Pietsch et al^23^ describe their process of predicting placental insufficiency by training and evaluating their AI algorithm on 108 3T MRI placental datasets comprised of free-breathing, rapid T2* relaxometry sequences. Within this dataset, 20 pregnancies were known to have a diagnosis of pre-eclampsia and/or fetal growth restriction (termed ‘high risk’ by the authors). The study pipeline involved an initial automatic segmentation of the placenta using a U-Net algorithm architecture, followed by a Gaussian process regression model to characterise placental maturation and health. Automatic segmentation was found to have a low DICE coefficient of 0.58, although the mean T2* values within the segmented placentas were comparable to human readers (0.986 Pearson Correlation Coefficient). A placental health prediction score was then calculated based on the mean T2* data generated by the segmentation method and clinical outcome details (*e.g.,* gestational age, neonatal and maternal outcomes) to generate a score. Z-scores were then compared between the high risk and low risk patient groups and when applied to an unseen dataset of 42 MRI cases (1.5T MRI, T2* sequences, six pre-eclampsia cases), and the automated pipeline of placental segmentation with derived placental health score was able to demonstrate consistently reduced Z scores (*Z* < −1.5) in those with pre-eclampsia compared to normal controls. Similarly, an algorithm by Dahdouh et al^42^ was able to use the shape and textural features of the placenta trained on 1.5T MRI studies (T2 sequences) in 50 patients (34 with fetal growth restriction) to predict restricted growth with a reported 86% accuracy. The reported birth weight estimations derived by the algorithm based on placental features was reportedly 0.3 ± 13.4% (mean percentage error ± s.d.) for healthy fetuses and −2.6 ± 15.9% for those with restricted growth.. Another study has shown promising results in predicting gestational age after the first trimester, when ultrasound is no longer accurate.^38^ In this study, 184 fetal examinations were collated, performed on 1.5T MRI, using T2 sequences of the fetal brains ranging between 14–41 weeks gestation. Reference standard for gestational age was the mother’s last menstrual period and ultrasound measurements from first trimester as reference. The authors found that there was a high concordance correlation between the model prediction for gestational age and the reference standard (pc = 0.964).

One interesting study was able to predict which fetuses would require post-natal intervention for ventriculomegaly to aid in surgical planning at an accuracy of up to 91%.^41^ Another novel use of AI tools was been reported by Torrents-Berrana et al,^25^ who described a role for their segmentation and reconstruction algorithm by creating a fully virtual simulation environment for clinician training in fetoscopic laser surgery. This is a high-risk procedure used in twin-to-twin transfusion syndrome and the AI-generated simulation platform not only provided a safe educational environment for trainees to practice their skills, but also allowed for pre-operative planning for the soundest entry point and approach for experienced surgeons.

### Miscellaneous

Finally, a significant limitation in generating AI algorithms for fetal MRI continues to be a lack of large datasets with multiple MRI sequences degraded by artefact. A solution to this is presented in a feasibility study by Torrents-Barrena et al^10^ where they use a ‘Generative Adversarial Network’ (GAN) to help create synthetic fetal MRI sequences in three different anatomical planes. They found that among the 384 simulated fetal MRI slices created, approximately 95.1% of them were considered ‘realistic’, with those in the sagittal plane most challenging for the GAN to recreate (with the uterus and maternal spine frequently appearing misaligned). Nevertheless, this pipeline and technique holds promise for the future where larger datasets are needed for training, improving and refining automated segmentation tools without the need for acquiring more imaging data or potentially compromising patient data privacy through data transfer across multiple centres.

## Discussion

In this article, we found that a small number of research articles explored the ways in which AI could be applied to fetal MRI; however these were mostly for fetal brain imaging and for data post-processing (*e.g.,* organ segmentation) than for diagnosis. A variety of use-cases are presented, some for classification of disease, prognostication and automated fetal biometric measurements. In general, the datasets used for training and testing fetal AI tools were quite small with less than 300 cases in total (compared to several thousands of cases in other radiological studies); with many datasets originating from only one institution. Most of the studies included in this review were early feasibility or pilot studies and at present there are issues regarding the generalisability of these early results, particularly if these will be applied to other centres, countries, or scanner manufacturers.

There is therefore a need for increased international collaboration regarding data sharing, multicentric databases and stronger working relationships to be formed between scientists and clinicians to ensure that the research remains clinically relevant and useful for a wider population. In the future, articles regarding the cost-benefit and cost-effectiveness of AI tools for fetal imaging should be conducted, as without this information uptake of such tools by hospital departments may be delayed if workflow efficiencies cannot be justified to commissioning bodies.

In contrast to the limited studies exploring AI tools for fetal MRI, the literature regarding applications of AI tools for fetal ultrasound imaging is abundant. This is probably due to the more widespread use of obstetric ultrasound in clinical practice and consequently the larger datasets available for analysis and training neural networks. Most of the fetal ultrasound literature similarly concentrates around the identification of various fetal anatomy and the automated acquisition of standardised biometric data for diagnosis, disease classification and workflow improvement.^48^ As examples, Ryou et al^49^ proposed an image processing solution based on 3D ultrasound, which contained segmentation of the fetus, estimation of standard biometry views, automatic measurements of biometry, and detection of fetal limbs. Others have used semi-automated AI tools to accurately estimate nuchal thickness^50^ and correlating ventricular septal wall thickness with gestational age in fetal echocardiography.^51^ In order to promote clinically useful AI algorithm development and testing for fetal MRI, it will be important for tertiary referral centres to each develop a research imaging database for federated learning and a streamlined method for anonymisation of their data and upload to secure research platforms for improved multicentric collaboration. One barrier to this is time and personnel as many institutions lack the expertise of data scientists, as well as differences amongst various centres regarding their patient confidentiality and local information governance regulations.^52–54^

From our scoping review and personal reflections, we believe there are several gaps which may provide fruitful avenues for further research relating to fetal MRI and AI tools. In general, we noted a lack of studies that assessed how natural language processing (NLP), could be used to communicate significant findings to clinicians (*e.g.,* automated emailing of reports with abnormalities) or how this could be used to enhance clinical governance activities (*e.g.,* mining for information from the patient records, radiology reports and DICOM metadata for a faster search and larger datasets for audit and research projects)^55^ and for education (*e.g.,* identifying rare and interesting cases for a teaching library). Furthermore, only a very small number of imaging classification studies had been published to aid fetal MRI radiology diagnosis and prognostication and few publications helped to provide real-world evidence of improved clinical workflow and efficiency in practice.

It is unlikely that the algorithms reported in this article will be available for clinical usage in the near future, as generalisation to different patient populations across different scanner manufacturers, sequence parameters and MRI field strengths will be major limiting factors. Given the high stakes and implications from inaccurate fetal imaging results, it will be very important that any new technology is shown to be robust before routine usage, and work on patient acceptability should be sought. Nonetheless, the help of AI algorithms for better placental evaluation may be one of the more feasible applications for translation into clinical practice and it will be fascinating to see how these use cases develop.

In this scoping review, there were some limitations. Due to the high output of articles relating to AI in the current climate, it is likely that by the time of publication we have neglected to include some other recent articles that were in press but not yet available at time of our literature search. Despite this, we tried to incorporate as many relevant recent articles via the Google Scholar search as possible to capture those on open source/pre-publication websites and performed our search criteria twice. Secondly, our search strategy did not include all available medical research databases, but instead focussed on the most popular ones where we believed there to contain the highest yield of cutting-edge AI research. Future literature reviews, particularly focussing on specific aspects of fetal MRI imaging using AI, should try to incorporate databases (*e.g.,* EMBASE, Scopus, arXiv) to gather a more comprehensive range of possible relevant articles.

In conclusion, the current climate presents many opportunities for collaboration between doctors, patients, and computer scientists and to produce clinically useful tools. Some of these may help to improve the quality and uptake of fetal MRI imaging in more centres, alleviating some of the struggles with image acquisition that is currently being faced. It is however important that future tools, particularly those that focus on clinical diagnosis and prognostication, are carefully validated and that larger datasets with varied pathologies, gestations and across different ethnic backgrounds are available for rigorous testing.
