# Supplementary material for: Artificial intelligence applied to fetal MRI: A scoping review of current research
Source: Br J Radiol. 2022 Mar 16;96(1147):20211205. doi: 10.1259/bjr.20211205 (PMC10321262; doi:10.1259/bjr.20211205)
Supplement: Supplementary Material 1. [file bjr.20211205.suppl-01.docx]

**Supplementary File**

Search in MEDLINE (PubMed interface), performed 16 September , 2021:

| # | Search Terms | Results |
| --- | --- | --- |
| 1 | "fetal"[Title/Abstract] OR "fetus"[Title/Abstract] OR "embryo"[Title/Abstract] OR "embryonal"[Title/Abstract] OR ("intrauterine"[Title/Abstract] OR "antepartum"[Title/Abstract] | 458,366 |
| 2 | "artificial intelligence"[Title/Abstract] OR "ai"[Title/Abstract] OR "deep-learning"[Title/Abstract] OR "deep-learning"[Title/Abstract] OR "machine learning"[Title/Abstract] OR "neural network"[Title/Abstract] | 139,705 |
| 3 | "MRI"[Title/Abstract] OR "imaging"[Title/Abstract] OR "radiology"[Title/Abstract] | 1,058,917 |
| 4 | #1 AND #2 | 1,699 |
| 5 | #4 AND #3 | 140 |
| 6 | #5 AND English[lang] | 138 |

Search in MEDLINE (PubMed interface), performed 26 January , 2022:

| # | Search Terms | Results |
| --- | --- | --- |
| 1 | "fetal"[Title/Abstract] OR "fetus"[Title/Abstract] OR "embryo"[Title/Abstract] OR "embryonal"[Title/Abstract] OR ("intrauterine"[Title/Abstract] OR "antepartum"[Title/Abstract] | 464,545 |
| 2 | "artificial intelligence"[Title/Abstract] OR "ai"[Title/Abstract] OR "deep-learning"[Title/Abstract] OR "deep-learning"[Title/Abstract] OR "machine learning"[Title/Abstract] OR "neural network"[Title/Abstract] | 99,584 |
| 3 | "MRI"[Title/Abstract] OR "imaging"[Title/Abstract] OR "radiology"[Title/Abstract] | 1,089,911 |
| 4 | #1 AND #2 | 566 |
| 5 | #4 AND #3 | 103 |
