# Supplementary material for: Artificial intelligence applied to fetal MRI: A scoping review of current research
Source: Br J Radiol. 2022 Mar 16;96(1147):20211205. doi: 10.1259/bjr.20211205 (PMC10321262; doi:10.1259/bjr.20211205)
Supplement: Supplementary Material 2. [file bjr.20211205.suppl-02.docx]

**Identification of studies via databases and registers**

**Records identified:**

MEDLINE (n = 148)

Grey Literature (n = 6)

**Identification**

**Records screened & assessed for eligibility**

(n = 154)

**Reports excluded (n = 115)**:

Not MRI related (n = 47)

Not AI related (n = 9)

Not fetal related (n = 19)

Basic science/animal study (n = 32)

Editorial/ review articles (n = 8)

**Screening**

**Studies included in review**

(n = 39)

**Included**

*From:*  Page MJ, McKenzie JE, Bossuyt PM, Boutron I, Hoffmann TC, Mulrow CD, et al. The PRISMA 2020 statement: an updated guideline for reporting systematic reviews. BMJ 2021;372:n71. doi: 10.1136/bmj.n71
